# Tele-monitoring reduces exacerbation of COPD in the context of climate change–a randomized controlled trial

**DOI:** 10.1186/1476-069X-12-99

**Published:** 2013-11-21

**Authors:** Melissa Jehn, Gavin Donaldson, Bahar Kiran, Uta Liebers, Klaus Mueller, Dieter Scherer, Wilfried Endlicher, Christian Witt

**Affiliations:** 1 Division of Pneumological Oncology and Transplantology, Charité Universitätsmedizin Berlin, Berlin, Germany; 2 Centre for Respiratory Medicine, Royal Free & UCL Medical School, London, UK; 3 Institut for Social Economy, Leibniz-Zentrum für Agrarlandschaftsforschung e.V.; on behalf of the KLIMZUG Research Group, Berlin, Germany; 4 Department of Ecology, Technische Universität Berlin; on behalf of the UCaSH Research Unit, Berlin, Germany; 5 Geography Department, Humboldt-Universität zu Berlin; on behalf of the KLIMZUG Research Group, Berlin, Germany

**Keywords:** Climate change, Telemedicine, Heat stress, Exacerbation frequency, Activity monitoring

## Abstract

**Background:**

A home based tele-monitoring system was developed to assess the effects of heat stress (days > 25°C) on clinical and functional status in patients with chronic obstructive pulmonary disease (COPD).

**Methods:**

Sixty-two COPD patients (GOLD II–IV) were randomized into a tele-monitoring Group (TG, N = 32) or Control Group (CG, N = 30). Tele-monitoring included 1) daily clinical status (COPD Assessment Test-CAT), 2) daily lung function and 3) weekly 6-minute walk test (6MWT). Duration of monitoring lasted a total of nine months (9 M).

**Results:**

From June 1^st^–August 31^st^ 2012, 32 days with heat stress (29.0 ± 2.5°C) were recorded and matched with 32 thermal comfort days (21.0 ± 2.9°C). During heat stress, the TG showed a significant reduction in lung function and exercise capacity (FEV_1_% predicted: 51.1 ± 7.2 vs. 57.7 ± 5.0%; *P* <0.001 and 6MWT performance: 452 ± 85 vs. 600 ± 76 steps; *P* <0.001) and increase in CAT scores (19.2 ± 7.9 vs. 16.2 ± 7.2; *P* <0.001).

Over summer, significantly fewer TG patients suffered exacerbation of COPD compared to CG patients (3 vs. 14; *P* = 0.006). Over entire 9 M follow-up, the TG group had fewer exacerbations compared to CG (7 vs. 22; *P* = 0.012), shorter cumulative hospital stay (34 vs. 97 days) and 43% fewer specialist consultations (24. vs. 42; *P* = 0.04).

**Conclusion:**

Heat stress affects clinical and functional status in COPD. Tele-monitoring reduces exacerbation frequency and health care utilization during heat stress and other periods of the year.

**Trial registration:**

DRKS-ID: DRK00000705.

## Background

The effects of climate change and global warming on human health, in particular in patients with lung disease, are poorly understood at present. The lung is of particular interest in this context, because it can be seen as the portal organ of the environment. For this reason, chronic obstructive pulmonary disease (COPD) is an ideal disease model to help us comprehend the dynamics between human health and climate change [1].

The incidence and prevalence of COPD is rapidly increasing and experts predict it to be the third leading cause of mortality worldwide [2]. Depending on disease severity, patients suffer from mild to severe airflow limitations associated with an accelerated decline related lung function [3]. One of the most prominent causes for hospital admissions in older patients is acute exacerbation of COPD (AECOPD) and previous research suggests that this condition is aggravated by extreme temperatures [2,4,5]. AECOPD is characterized by a sudden worsening of respiratory symptoms such as shortness of breath, sputum production, coughing and wheezing. Clinical manifestations of AECOPD include increased airway inflammation, reduced expiratory air flow and worsening of gas transfer [6].

There is abundant epidemiological data showing increased morbidity and mortality rates during heat waves in patients with respiratory diseases, including COPD [7-10]. Nevertheless, little is known about the clinical and lung functional consequences of heat stress in COPD [9]. Elderly patients (> 65 years of age) with COPD experience a heightened medical vulnerability to heat stress, because lung perfusion and bronchial mucosa are negatively affected by hot air loaded with air pollutants [11]. In addition, the control of cutaneous blood flow and the ability to sweat is compromised in the elderly, as well as an inability to perceive changes in heat and thus adjust behavioral responses i.e. by increasing fluid intake [12,13]. Heat intolerance is further augmented by chronic medication use and additional comorbidities such as cardiovascular disease [7,10]. During heat stress, dilation of peripheral bloods vessels requires the heart to work harder to maintain central blood pressure, a problem which is possibly worse with heart failure, a common co-morbidity in patients with COPD [14]. The increased cardiac work and tachycardia may increases the sensations of breathlessness and make the perception of exacerbation more likely [15-17]. Evidence has shown that elderly patients with COPD are at greater risk for exacerbation of COPD if the following social and/or working factors apply: 1) living alone, 2) confined to the house, 3) have poor social contact, 4) poverty, 5) no access to transportation, 6) no green space and 7) no working fan or air conditioning use [11].

According to climate change projections, the risk for heat exposure will continue to increase due to the increased frequency, longer duration and greater intensity of hot spells [18,19]. Targeted adaptation strategies are needed in order to counteract upcoming health hazards, in particular in patients with advanced COPD. The multidisciplinary German Federal Research Program KLIMZUG, “Klima in Regionen zukunftsfähig gestalten” aims to investigate adaptation strategies to climate change in different regions of Germany. One of the main working packages of this project is dedicated to climate change and public health, and in particular, evaluation of vulnerability to heat stress of patients with chronic obstructive pulmonary disease (COPD) in Berlin, Germany, a metropolitan area surrounded by the countryside of Brandenburg (Trial registration: URL: https://drks-neu.uniklinik-freiburg.de/drks_web/; ID: DRK00000705). Primary and secondary study endpoints of this project include 1) determination of how heat stress exposure influences clinical status and functional capacity and 2) development and evaluation of a home based tele-monitoring system for COPD patients to investigate whether it reduces the risk of exacerbation during and outside periods of heat stress.

## Methods

Patient recruitment took place between Jan–April 2012 with study duration of nine months. To be eligible for the study, patients had to be diagnosed with COPD Stage II-IV on the basis of a clinical history such as smoking status, physical examination and meet the post-bronchodilator spirometric criteria according to the GOLD guidelines (FEV1 < 80% predicted & FEV1/FVC ratio <0.7). Spirometry was performed by trained lung function technicians according to American Thoracic Guidelines [6]. Patients also had to have at least one exacerbation during the previous year, be ≥ 40 years of age and clinically stable for the four weeks prior to inclusion. Patients were excluded from the study if they suffered from 1) asthma, 2) required long-term oxygen therapy, 3) had severe heart, liver or kidney disease, 4) any end stage malignant disease with life expectancy of less than six months, 5) were listed for a lung transplant, 6) had severe depression, 7) were residents in a nursing home, 8) had any physical disabilities limiting them from performing a six minute walk test (6MWT) and/or 9) were mentally disabled.

Acute exacerbation of COPD (AECOPD) was defined according to significant worsening of respiratory symptoms requiring change in medication (oral corticosteroids and/or antibiotics) and the presence of at least one of the following items: 1) increased dyspnea, 2) increase in the amount of sputum production and 3) change in sputum purulence [20]. Primary diagnosis of acute exacerbation of COPD was done by the treating physician in the emergency room and later confirmed by the study physician. All exacerbations in this study analysis were defined as a minimum of ≥ 24 hour hospital stay–duration time.

Patients were randomized into either a Telemedicine group (TG) who participated in a tele-monitoring intervention or a control group (CG). Both groups also received usual care which was based on current guidelines for the treatment and management of COPD and included an initial baseline examination and regular follow-up visits at 3, 6, and 9 M. At these visits, a detailed medical history, current medication, duration of disease, co-morbidities, physical examination, height, quality of life (SGRQ [21]), modified Medical Research Council Dyspnoea Scale (mMRC), spirometry, CAT, and 6MWT distance assessed with a distance measuring wheel as well as the number of steps taken during the 6 minutes (6MWT step count) assessed via accelerometry were recorded. Patients were also asked about the number of visits to their primary care physician or lung specialist.

The tele-monitoringintervention included 1) daily assessment of clinical status by means of the COPD Assessment Test (CAT), 2) daily lung function testing (spirometry), and 3) a weekly six-minute walk test measured by accelerometry. Telemedicine group patients completed all three testing modalities in their home-based environment. Following completion of the tests, patient data were transmitted via a mobile network directly to the study center at the Charité University Hospital, in Berlin. Patients were asked to complete all measurements in the morning within a two-hour time window of their choice following inhalation of their usual bronchodilators.

Patients were thoroughly trained how to use all technical equipment during their baseline visit. A study nurse was available during regular business hours five days per week to provide first-line technical support in order to ensure correct handling of the devices and continuous data transmission. The study nurse also contacted patients if data was not sent during the agreed time window to remind patients to complete the tests if necessary; for safety reasons incoming data was reviewed by the study physician on a daily basis, however data was gathered in an observational manner.

The study was carried out according to the principles of the Declaration of Helsinki and approved by our local ethics committee “Ethikkommission Charité - Universitätsmedizin Berlin” (Ethic Number EA1/033/10). Written informed consent was obtained from all patients.

### Tele-monitoring system

1. COPD assessment test (CAT)

The CAT (© 2009 GlaxoSmithKline) is directly entered into the mobile medical assistant (PDA system, MMA 400, Aipermon GmbH & Co. KG, Munich, Germany) by the patients. This assessment test is composed of eight questions each containing a scale from 0 (= never) to 5 (= always). Question 1: asks coughing frequency, question 2: level of congestion (sputum), question 3: tightness in the chest, question 4: exercise induced dyspnea, question 6: degree of functional impairment during routine activities, question 7: quality of sleep, and question 8: level of energy.

2. Assessment of lung function

Lung function was measured with a hand held spirometer (Vitalograph asma-1 BT, Aipermon GmbH & Co. KG, Munich, Germany). Patients were asked to perform each measurement in a seated position, hold a maximal inhalation, place the plastic mouthpiece of the spirometer into the mouth and exhale as forcefully and as fast as possible for at least six seconds. The following lung function parameters are displayed on the screen in subsequent order: peak expiratory flow (PEF), forced expiratory volume in (ml) in one second (FEV_1_), forced expiratory volume in six seconds (FEV_6_) and forced expiratory flow 25–75% (FEF_25-75_). The patients were instructed to repeat this breathing maneuver three times in a row and the best of three measurements was then transmitted to the study center at the university hospital. Only FEV_1_ as a percentage of a normal value predicted from age, gender and height was used for statistical analysis.

3. Assessment of weekly exercise capacity

Exercise capacity was assessed in form of the six-minute walk test (6MWT) measured by a distance wheel (model: Draper Expert Measuring Wheel) and accelerometry (AiperMotion 300 PfH, Aipermon GmbH & Co. KG, Munich, Germany). The study nurse trained the patients in the test procedure, and instructed them to conduct the 6MWTs on their own in their outdoors environment using a flat walking path that would allow them to walk continuously for six minutes without interruption. To assure consistency, patients were asked to use the same walking path throughout the nine-month intervention period. The AiperMotion 300 device is a three dimensional accelerometer customized especially for recording data during the 6MWT with a “start 6MWT” button and automatic end of data recording after 6 minutes. The accelerometer is matchbox sized and worn on hip level attached to the belt via a pocket pouch. Data output includes the number of total steps taken, distance and walking speed in six minutes. Measurement accuracy of the device has been previously validated in patients with chronic heart failure under laboratory conditions as well as in a field based setting during which the validity of 6MWT steps was established to be a suitable measure of functional exercise capacity [22].

### Assessment of temperature data

Data regarding daily temperature in degree Celsius were obtained from the local weather station (Tempelhof, Berlin, Germany; maintained by the Deutscher Wetterdienst) and included daily temperature readings (Temp_mean_, Temp_max_, Temp_min_), air pressure (hPa) and humidity (%). This weather station is located 48 m above NN on 52.47° N 13.4° E. Days with Temp_max_ > 25°C were considered “heat stress days”, whereas days with Temp_max_ ≤ 25°C were regarded as “thermal comfort days”.

### Statistical analysis

Statistical analysis was performed using SPSS software (version 21.0, SPSS Inc.). Normally distributed data are reported as mean ± standard deviation (SD) or median ± .inter-quartile range, or as percentages for dichotomous variables. Differences were compared using two-tailed *t* test for normally distributed variables and Chi^2^-test for dichotomous variables. *P*-values less than 0.05 were considered statistically significant. From June 1^st^–August 31^st^ 2012, 32 heat stress days were recorded and matched with 32 thermal comfort days. Days with thermal comfort were randomly chosen during the respective summer months. Box blots were used to illustrate the dynamics between daily patient measurements and temperature data and dual axis bar plots were used to illustrate the influence of daily temperature on clinical parameters. Bar plots were also drawn to illustrate the total number of exacerbations requiring hospitalizations and outpatient consultation to the lung specialist and primary care physician. Only the intention to treat population was analyzed.

## Results

### Patient characteristics

Sixty-two patients with COPD were included in this current study analysis (TG, N = 32; CG N = 30) with moderate to very severe disease (GOLD II, N = 25; GOLD III, N = 25, GOLD IV, N = 12). Their mean age was 65.7 ± 10.3 years and 74% were men; most patients were taking a long acting anticholinergic agent (76%) and a long-acting beta-2 agonist/inhaled corticosteroid combination (71%). There were no statistically significant differences in baseline characteristics between the TG and the CG (see Table 1).

**Table 1:** Patient baseline characteristics (N = 62)

**Group**	**TG**	**CG**	***P*-Value**
**N**	32	30	
**Gender (male/female)**	26/6	22/8	.79
**Age (years)**	64.1 ± 10.9	69.1 ± 9.2	.27
**BMI (kg/m**^**2**^**)**	27.6 ± 6.7	27.0 ± 4.9	.87
**Resting SpO**_**2 **_**Saturation**	95.4 ± 1.7	94.3 ± 3.6	.095
**FEV**_**1 **_**(%)**	50.2 ± 15.0	52.6 ± 17.5	.731
**PEF (L/min)**	3.7 ± 1.23	4.4 ± 1.4	.194
**MMRC**	1.9 ± 1.1	1.2 ± 0.93	.125
**GOLD**	2.6 ± 0.8	2.5 ± 0.9	.83
**BODE**	3.2 ± 3.1	2.9 ± 3.0	.55
**SGRQ**	51.3 ± 18.7	48.7 ± 14.7	.56
**COPD Medication**			
**Long AC**	25 (78%)	22 (73%)	.33
**ICS/LABA**	23 (72%)	21 (70%)	.46

Characteristics of patient population; data is presented as Mean ± SD; group comparisons of measurements across between Tele-monitoring group (TG) and control group (CG) were done using a two tailed student’s t-test and χ^2^-test. BMI = Body Mass Index; SpO_2_ = resting oxygen saturation; FEV_1_ (L) = Forced Expiratory Volume in one second; PEF = Peak Expiratory Flow; MMRC = Modified Medical Research Council Dyspnea Scale; SGRQ = St. George Respiratory Questionnaire; Long AC = Long Acting Anticholinergic; ICS/LABA = fix combination of ICS/LABA.

### Telemedical patient data

#### Summer period (June 1^st^–August 31^st^)

**Figure 1:**
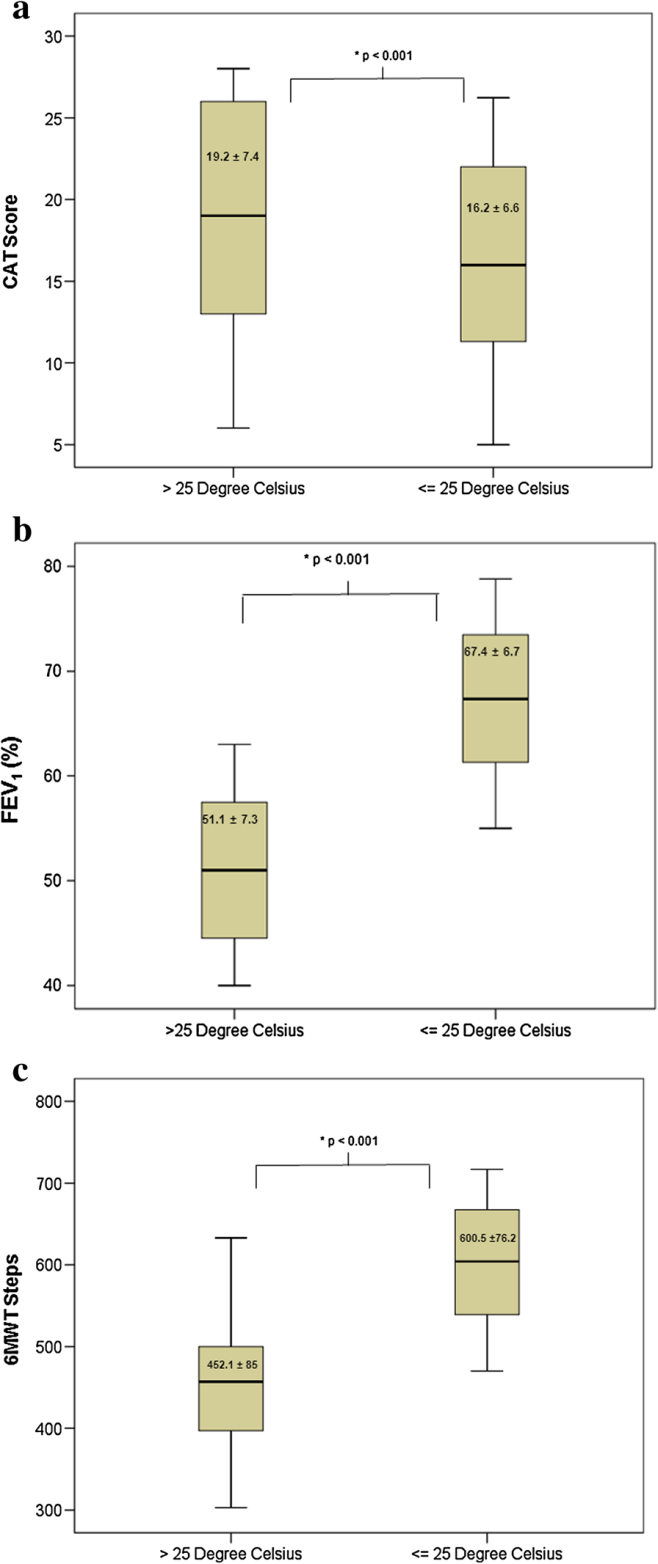
**Clinical and functional status on days with heat stress vs. thermal comfort.** Box plots showing **a)** CAT Score, **b)** FEV_1_ (%) and **c)** 6MWT steps on 32 days with Temp_max_ > 25°C compared to 32 days with Temp_max_ ≤ 25°C. Statistical significance is set at *P* < 0.05.

**Figure 2:**
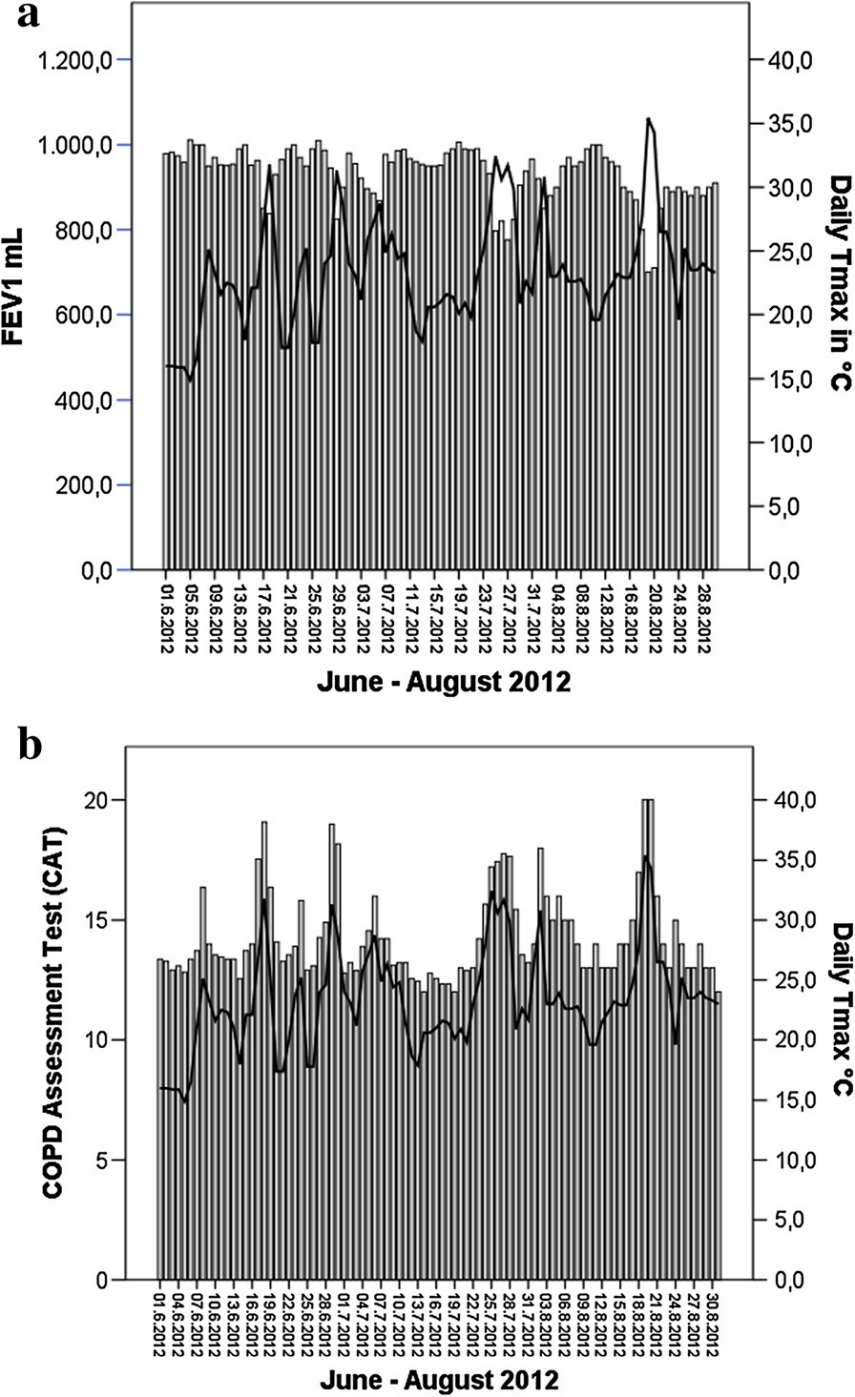
**Variation in daily clinical status and lung-function in response to outside temperature.** Dual axis box plots with the x-axis depicting **a)** FEV_1_ (ml) and **b)** CAT score. The y-axis indicates the days from June–31^st^ August and the z-axis (black line) shows daily temperature fluctuations.

Heat stress days with thermal discomfort as defined by the German Weather Service reached an average Temp_max_ of 29.0 ± 2.5°C. Thermal comfort days reached an average Temp_max_ of 21.0 ± 2.9°C. There was a significant difference (*P* < 0.001) in functional performance and clinical status on “heat stress days” versus “thermal comfort days”, with a marked decrease in FEV_1_ (Mean difference: –6.6 ± 6.1%) and 6MWT performance (Mean difference: -48.0 ± 80 steps), as well as a concomitant increase in CAT score (Mean difference: + 3.0 ± 7.6 points) (see Table 2 and Figure 1a-c). Day to day variations in clinical parameters in response to outside temperature fluctuations for the months June–August 2012 are depicted in Figure 2a and b.

**Table 2:** Remote patient data: “Heat stress” days vs. “Thermal comfort” days

**Temperature**	**Temp**_**max**_ **> 25°C**	**Temp**_**max**_ **≤ 25°C**	***P*-value**
N	32	32	
Temp_max_ °C	29.0 ± 2.5	21.0 ± 2.9	< 0.001*
Temp_variance_ K	13.5 ± 3.0	12.0 ± 2.4	0.017*
Air Humidity (%)	58.8 ± 13.7	60.8 ± 9.6	0.517
Air Pressure (hPa)	1008 ± 4.5	1009 ± 11.4	0.769
6MWT steps	452 ± 85	600 ± 76	< 0.001*
FEV_1_ (%)	51.1 ± 7.2	57.7 ± 5.0	< 0.001*
CAT score	19.2 ± 7.9	16.2 ± 7.2	< 0.001*

Remote patient data on days with heat stress vs. thermal comfort days. Data is expressed as means ± SD. Comparisons are made via paired student’s t-test; *statistical significance is set at *P* < 0.05; Please see Table 1 for all other abbreviations.

### Clinical outcome of study population

#### Summer period (June 1^st^–August 31^st^)

**Figure 3:**
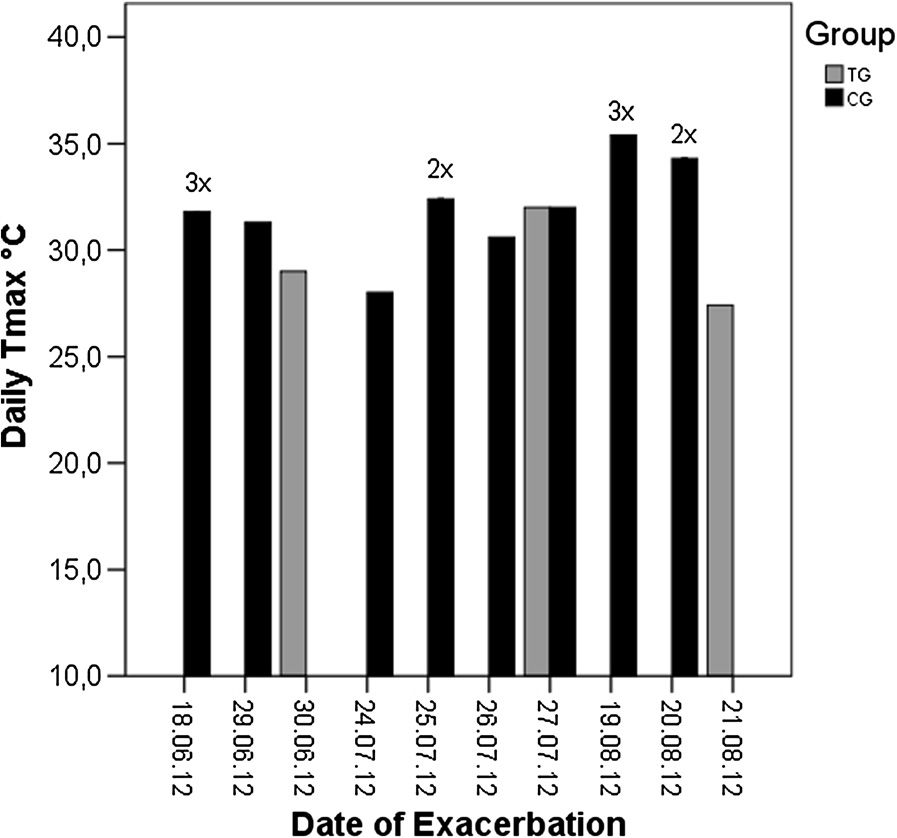
**Bar graph showing the number of exacerbation from June–August 2012 in the Telegroup (TG = grey boxes, N = 3) and the Control Group (CG = black boxes, N = 14) with corresponding date of event on the x-axis and daily T**_**max **_**in °C on the y-axis.**

Significantly fewer TG patients suffered exacerbation of COPD during the summer period compared to CG patients (3 for TG vs. 14 for CG; *P* = 0.006). Temp_max_ on days of exacerbation reached an average of 32.6 ± 2.0°C and atmospheric ozone levels of 130.3 ± 20.1 mg/m^3^ (see Figure 3). Patients that suffered exacerbation of COPD on heat stress days tended to be male, slightly older in age (mean age: 70 ± 5.6 years), have higher BMI (27.5 ± 3.3 kg/m^2^), lower FEV_1_% predicted (38.2 ± 8.1%) and higher number of co-morbidities (Charlson Index: 2.9 ± 1.0) than patients that suffered exacerbation during other months of the year but these differences were not statistically significant.

### Over 9-month follow-up

**Figure 4:**
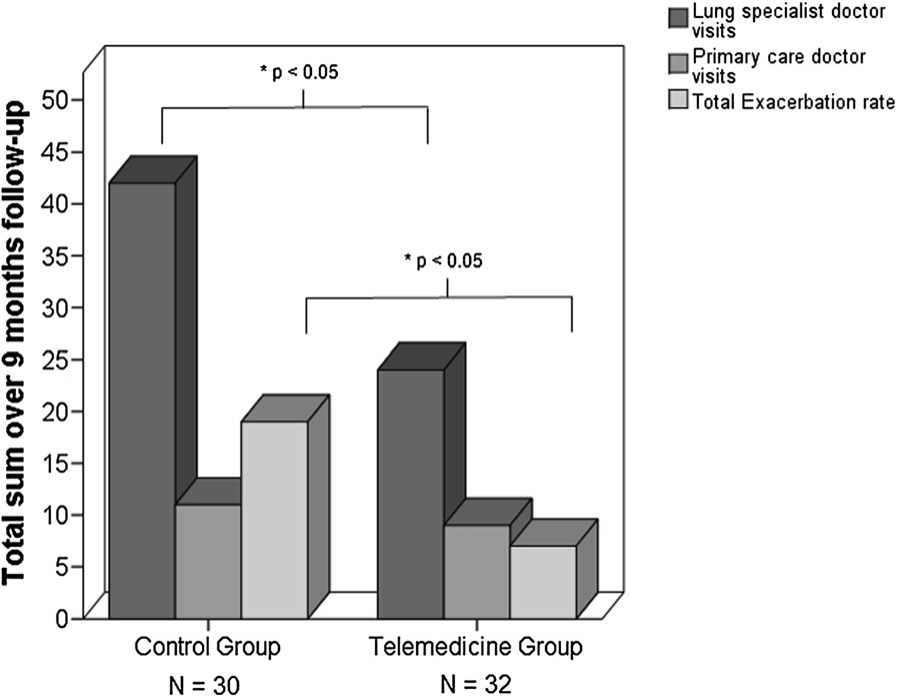
**Bar graph representing number of exacerbations (light grey), visits to the primary care physician (medium grey) and to the lung specialist (dark grey) in the Telegroup (TG, N = 32) versus the Control Group (CG, N = 30).** Statistical significance is set at *P* < 0.05.

Overall the TG showed a significantly lower number of exacerbation related hospital admissions over the 9 M follow-up time compared to the CG (7 for TG vs. 22 for CG; *P* = 0.012), significantly fewer visits to the lung specialist (24 for TG vs. 42 visits for CG; *P* = 0.042) and slightly fewer visits to the primary care physician (9 for TG vs. 11 visits for the CG; *P* = 0.76) (see Figure 4). In addition, patients in the TG spent significantly less cumulative time in hospital due to COPD complications compared to the CG (34 days versus 97 days).

Within each group, the TG experienced a significant improvement in clinical status between BL and 9 M follow-up (Mean Difference in CAT score: -2.9 ± 4.5; *P* = 0.04), in contrast to the CG group in whom clinical status worsened in this time (Mean Difference in CAT score: +4.4 ± 5.7; *P* = 0.013). The TG group also showed a significant improvement in 6MWT distance between BL and 9 M follow-up (Mean Difference in 6MWT: +87.0 ± 65.7 metres, *P* = 0.006), whereas the CG group showed no significant change (Mean Difference in 6MWT: +23.9 ± 70.3 metres, *P* = 0.23). Spirometry remained unchanged in both groups between BL and 9 M follow-up. Between both groups, only the change in CAT score was significantly different between BL and 9 M follow-up (*P* = 0.001). See Table 3.

**Table 3:** Baseline compared to 9 month Follow-up visit

**Group**	**Telegroup, N = 27**			***P*-value**	**Control group, N = 25**			***P*-value**
	**BL**	**9 M**	**Mean difference**		**BL**	**9 M**	**Mean difference**	
**6MWT steps**	522 ± 113	662 ± 86.4	140 ± 95.2	0.006*	553 ± 103	601 ± 88.7	48.3 ± 85.2	0.23
**6MWT distance**	377 ± 88.0	464 ± 60.5	87.0 ± 65.7	0.006*	377 ± 78.0	400 ± 72.4	23.9 ± 70.3	0.23
**FEV1%**	50.2 ± 15.0	52.7 ± 16.7	2.5 ± 5.2	0.10	52.6 ± 17.4	52.6 ± 19.8	-.07 ± 9.2	0.99
**CAT**	19.0 ± 6.9	16.0 ± 5.6	−2.9 ± 4.5	0.04*	17.6 ± 5.0	22.0 ± 6.9	4.4 ± 5.7	0.013*

Characteristics of patient population baseline (BL) compared to 9-month follow-up; data is presented as Mean ± SD; group comparisons of measurements across different Randomization Groups were done using a paired student’s t-test; *statistical significance is set at *P* ≤ 0.05. 6MWT = 6-minute walk test; FEV_1_ (%) = Forced Expiratory Volume in one second% predicted; CAT = COPD Assessment Test.

### Exacerbation vs. event free patients

Patients that suffered an exacerbation had significantly lower FEV_1_% at BL compared to patients that remained event free (44.2 ± 13.1% vs. 57.2 ± 20.2%, respectively; *P* = 0.045) and significantly lower BL exercise capacity (6MWT steps: 450 ± 87.3 steps vs. 537 ± 91.2 steps; *P* = 0.025). None of the other BL characteristics listed in Table 1 showed any significant difference between patients with exacerbation and patients that remained event free.

## Discussion

We have several findings to report from this study: 1) Clinical status, lung function and exercise capacity are negatively affected by heat stress in patients with COPD. By means of continuous tele-monitoring we were able to show significant worsening in all three parameters on heat stress days compared to thermal comfort days during the summer period. 2) Heat stress aggravates COPD symptoms putting patients at greater risk for exacerbation of COPD. Tele-monitoring seems to reduce the risk of exacerbation during the summer period. 3) Continuous tele-monitoring improves clinical and functional status of COPD patients over time and reduces the frequency of exacerbation and health care utilization during all periods of the year.

Previous investigators have reported on the impact of heat stress in elderly COPD patients, [10,11,23], however these studies have focused primarily on epidemiological data in terms of morbidity and mortality rates [1,9,24,25]. To the best of our knowledge, this is the first study that has looked at changes in physiologic parameters in response to outside temperature in patients with COPD. With the help of continuous tele-monitoring during the summer months, we were able to investigate the direct effect heat stress has on health status in our patient cohort. Our data show that heat stress negatively impacts patients’ functional capacity and clinical status including exacerbation of COPD. Our data also show that tele-monitoring reduced the incidence of exacerbation of COPD, both in the summer and over the 9 month study period. Although we cannot be sure that summer exacerbations are directly caused by heat stress in our patient cohort, we did observe that tele-monitoring reduces the risk of exacerbation, heat related or not. Therefore it seems beneficial to implement tele-monitoring in vulnerable patient groups during prolonged heat exposure.

A recent study by Jensen et al. also looked at the overall benefits of tele-monitoring in COPD patients and reported similar findings to ours in terms of clinical outcome and disease management [26]. We agree with the conclusions made by Jensen et al., in that the overall improvements seen in COPD patients are to the most part attributable to the continuous task of home based testing. It brings patients into closer contact with their disease by enhancing disease awareness and self-management skills. Other studies have also demonstrated a significant reduction in hospital utilization in COPD patients using self-management programms [27-29]. A study by Vitacca et al. was able to show a 50% reduction in health care expenditure per COPD patient managed by tele-monitoring compared to patients receiving usual care only [30].

What makes our tele-monitoring system novel in comparison to other telemedical studies in COPD is that we included the element of weekly exercise testing via accelerometry. Our data show that this approach is feasible and safe in patients with COPD. We still believe that improvements in our TG patients are driven by enhanced disease awareness on the patients’ side; however, it is possible that the weekly 6MWT elicited a slight “exercise training” effect, as observed with pulmonary rehabilitation, in otherwise sedentary COPD patients. It is also possible that the weekly 6MWT promoted participation in other recreational activities in our patient cohort thereby putting patients more in touch with their physical boundaries. Studies have shown that regular physical activity increases exercise tolerance, reduces dyspnea, fatigue, anxiety and depression in patients with COPD [31]. In addition regular exercise has also shown to reduce costs associated with the disease by decreasing use of the health care system, in particular unplanned hospitalizations due to exacerbation of COPD [32].

Limitations to this study include the small patient numbers but time and resource constraints did not allow us to enroll more patients. Moreover, our primary study goal was to evaluate the impact of urban heat stress on patients with COPD. The strength of this study lies in the use of tele-monitoring to obtain date stamped clinical status and functional performance data during both heat stress and thermal comfort days. Our COPD patients were of stages GOLD II–IV and sufficiently ambulatory to perform a 6MWT, thus our findings should not be extrapolated onto COPD patients who are not fully ambulatory, because they use oxygen therapy or who have co-morbidities such as osteoarthritis or rheumatoid arthritis.

According to a report from the Center for Health and the Global Environment at Harvard Medical School, there will be an increase in the number, duration and intensity of extreme weather events, including heat waves [33]. The changing climate appears to show an increase in temperature variance leading to more record hot days. This will cause clinical status and quality of life to deteriorate in patients with COPD. Adaptation strategies must be in place to protect vulnerable patients at risk for heat stress in order to reduce poor outcome. These include, but are not limited to, tele-monitoring, patient schooling about proper adaptive behavior during and outside of heat stress, as well as necessary adjustments in pharmacological therapy. These findings are preliminary and must be confirmed in a larger patient population.

## Conclusion

Heat stress negatively impacts clinical and functional status in patients with COPD and makes patients more vulnerable for disease related morbidity. Tele-monitoring reduces exacerbation frequency and should be implemented on top of regular patient care during periods of prolonged heat exposure.

## Abbreviations

COPD: Chronic obstructive pulmonary disease; GOLD: Global initiative for obstructive lung disease; MMRC: Modified medical research council dyspnea scale; SGRQ: Saint georges respiratory questionnaire; FEV1: Forced expiratory volume in one second; PEF: Peak expiratory flow; BMI: Body mass index; TG: Telegroup; CG: Controlgroup; 6MWT: 6-Minute walk test; CAT: COPD assessment test; SpO2: Resting oxygen saturation; BODE: Body-mass index, airflow obstruction, dyspnea, and exercise capacity index; Long AC: Long acting anticholinergic; ICS/LABA: Fix combination of ICS/LABA.

## Competing interests

The authors declare that they have no competing interests.

## Authors’ contributions

ML coordinated and supervised the study, performed the statistical analysis and drafted the manuscript; GD contributed to the statistical analysis, discussion of results and draft of the manuscript; MG helped coordinate the study and draft of the manuscript; UL contributed to study design, coordination and draft of the manuscript; KM provided expert advice on meteorological data and helped draft the manuscript; DS provided expert advice on meteorological data and helped draft the manuscript; WE helped conceive and design the study, provided expert advice on meteorological data and helped draft the manuscript; CW conceived, designed and supervised the study, contributed to the discussion of results and draft of the manuscript. All authors read and approved the final manuscript.
